# The Biological Effects of Smoking on the Formation and Rupture of Intracranial Aneurysms: A Systematic Review and Meta-Analysis

**DOI:** 10.3389/fneur.2022.862916

**Published:** 2022-07-12

**Authors:** Hanbin Wang, Luxuan Wang, Jiyue Wang, Lijian Zhang, Chunhui Li

**Affiliations:** ^1^School of Clinical Medicine, Affiliated Hospital of Hebei University, Hebei University, Baoding, China; ^2^Department of Neurology, Affiliated Hospital of Hebei University, Hebei University, Baoding, China; ^3^Department of Gastroenterology, Baoding No. 1 Central Hospital, Baoding, China; ^4^Postdoctoral Research Station of Neurosurgery, Affiliated Hospital of Hebei University, Hebei University, Baoding, China; ^5^Key Laboratory of Precise Diagnosis and Treatment of Glioma in Hebei Province, Affiliated Hospital of Hebei University, Hebei University, Baoding, China; ^6^Department of Neurosurgery, Affiliated Hospital of Hebei University, Hebei University, Baoding, China

**Keywords:** intracranial aneurysm, ruptured, smoking, nicotine, morphology

## Abstract

**Background:**

Aneurysms of the cerebral vasculature are relatively common, which grow unpredictably, and even small aneurysms carry a risk of rupture. Rupture of intracranial aneurysms (IA) is a catastrophic event with a high mortality rate. Pieces of evidence have demonstrated that smoking is closely related to the formation and rupture of IA. However, the biological effect of smoking cigarettes on the formation and rupture of IA is still underrepresented.

**Methods:**

The study protocol was prospectively registered in PROSPERO, registration number CRD42020203634. We performed a systematic search in PubMed and CNKI for studies exploring the biological effects of smoking on intracranial aneurysms published up to December 2021, and all studies were included in the analysis. The RevMan software was used for data analysis.

**Results:**

A total of 6,196 patients were included in 14 original articles in this meta-analysis. The risk of ruptured IA in the current smoking group was significantly higher than that in the non-smoking group, with statistical significance (RR_total_ = 1.23, 95% *CI*: 1.11–1.37). After heterogeneity among cohorts was removed by the sensitivity analysis, there was still a statistically significant difference in the risk of ruptured IA between the smoking and non-smoking groups (RR total = 1.26, 95% *CI*: 1.18–1.34). There was no statistically significant difference in the risk of ruptured IA between the former smoking (smoking cessation) group and the non-smoking group (RR_total_ = 1.09, 95% *CI*: 0.50–2.38). After heterogeneity among cohorts was removed by sensitivity analysis, there was still no statistically significant difference in the risk of ruptured IA between the former smoking (smoking cessation) group and the non-smoking group (RRtotal = 0.75, 95% *CI*: 0.47–1.19). The risk of the ruptured IA in the current smoking group was significantly higher than that in the former smoking (smoking cessation) group, with a statistically significant difference (RR_total_=1.42, 95%*CI*: 1.27–1.59).

**Conclusion:**

Although the biological effects of smoking on the formation and rupture of IA are unknown, this study suggests that current smoking is a risk factor for ruptured IA. Quitting smoking is very important for patients with IA.

## Introduction

An intracranial aneurysm (IA) is a partial expansion of cerebral blood vessels caused by the local abnormalities of blood vessels. The incidence of IA is 3–5% in the general population (1), and 85% of subarachnoid hemorrhage (SAH) is due to the rupture of IA, which could be seriously hazardous to health (2). Since most IAs are diagnosed after its rupture, it is of great importance to screen its risk factors.

At present, the diagnosis of IA in clinical settings mainly relies on neuroimaging technology (3). In addition, the use of MRI and CT as diagnostic tools has increased over the past 20 years as the quality of intracranial imaging techniques improved, and unruptured aneurysms have been detected with increasing frequency (4). Although the annual risk of ruptured incidental IA is relatively low (5), the prognosis of SAH caused by IA rupture is poor, with the 1 month case fatality rate still as high as 35% (6). About one-third of survivors require lifelong care, and another one-third have residual cognitive impairment that affects functional status and quality of life (7). Thus, preventing rupture of IA is vital. However, surgical clipping and endovascular intervention to prevent aneurysm rupture also have associated hemorrhagic and ischemic risks, which may exceed the natural risk of rupture (estimated total morbidity and mortality in the first month is 10–14%) (8, 9). Therefore, the prevention of IA by identifying and managing the modifiable risk factors, rather than surgical clipping and intravascular intervention, has a significant clinical and social value (10).

Many pieces of literature have reported that risk factors for the formation and rupture of IA include age, female sex, hypertension, and smoking. Among them, smoking is the most easily controlled risk factor (11). A previous studies has documented that cigarette smoking was significantly associated with the growth and rupture of IA (12). However, the mechanisms by which smoking causes the formation and rupture of IA are unclear. In the present meta-analysis, we aimed to determine the relationship between current smoking, former smoking (smoking cessation), and non-smoking and the risk of ruptured IA. Further, we summarized the potential biological effects of smoking on the formation, growth, and rupture of IA, which could be helpful in studying the pathophysiological mechanism of smoking and IA formation and rupture.

## Materials and Methods

### Inclusion and Exclusion Criteria

The inclusion criteria were as follows: (1) prospective and retrospective cohort studies; (2) research related to smoking and IA; and (3) availability of complete data on smoking and IA or data that can be extracted or calculated from the articles. The exclusion criteria were as follows: (1) studies with nonhuman experiments; (2) studies unrelated to smoking and IA; (3) repeated articles or data; and (4) articles of the type abstract, letter, editorial, expert opinion, review, case report, or laboratory study.

### Search Strategy

We searched PubMed and CNKI for studies on the relationship between smoking and IA published as of December 2021. The search formula is as follows: (((intracranial aneurysm[Title/Abstract]) OR (Aneurysms, Intracranial[Title/Abstract]) OR (Intracranial Aneurysms[Title/Abstract]) OR (Aneurysm, Intracranial[Title/Abstract]) OR (Aneurysm, Anterior Communicating Artery[Title/Abstract]) OR (Anterior Communicating Artery Aneurysm[Title/Abstract]) OR (Aneurysm, Basilar Artery[Title/Abstract]) OR (Aneurysms, Basilar Artery[Title/Abstract]) OR (Artery Aneurysm, Basilar[Title/Abstract]) OR (Artery Aneurysms, Basilar[Title/Abstract]) OR (Basilar Artery Aneurysms[Title/Abstract]) OR (Basilar Artery Aneurysm[Title/Abstract]) OR (Aneurysm, Middle Cerebral Artery[Title/Abstract]) OR (Middle Cerebral Artery Aneurysm[Title/Abstract]) OR (Aneurysm, Posterior Cerebral Artery[Title/Abstract]) OR (Posterior Cerebral Artery Aneurysm[Title/Abstract]) OR (Berry Aneurysm[Title/Abstract]) OR (Aneurysm, Berry[Title/Abstract]) OR (Aneurysms, Berry[Title/Abstract]) OR (Berry Aneurysms[Title/Abstract]) OR (Brain Aneurysm[Title/Abstract]) OR (Aneurysm, Brain[Title/Abstract]) OR (Aneurysms, Brain[Title/Abstract]) OR (Brain Aneurysms[Title/Abstract]) OR (Cerebral Aneurysm[Title/Abstract]) OR (Aneurysms, Cerebral[Title/Abstract]) OR (Cerebral Aneurysms[Title/Abstract]) OR (Aneurysm, Cerebral[Title/Abstract]) OR (Giant Intracranial Aneurysm[Title/Abstract]) OR (Aneurysm, Giant Intracranial[Title/Abstract]) OR (Aneurysms, Giant Intracranial[Title/Abstract]) OR (Giant Intracranial Aneurysms[Title/Abstract]) OR (Intracranial Aneurysm, Giant[Title/Abstract]) OR (Intracranial Aneurysms, Giant[Title/Abstract]) OR (Mycotic Aneurysm, Intracranial[Title/Abstract]) OR (Aneurysm, Intracranial Mycotic[Title/Abstract]) OR (Aneurysms, Intracranial Mycotic[Title/Abstract]) OR (Intracranial Mycotic Aneurysm[Title/Abstract]) OR (Intracranial Mycotic Aneurysms[Title/Abstract]) OR (Mycotic Aneurysms, Intracranial[Title/Abstract]) OR (Aneurysm, Anterior Cerebral Artery[Title/Abstract]) OR (Anterior Cerebral Artery Aneurysm[Title/Abstract]) OR (Aneurysm, Posterior Communicating Artery[Title/Abstract]) OR (Posterior Communicating Artery Aneurysm[Title/Abstract])) AND ((Smoking[Title/Abstract]) OR (Smoking Behaviors[Title/Abstract]) OR (Behavior, Smoking[Title/Abstract]) OR (Behaviors, Smoking[Title/Abstract]) OR (Smoking Behavior[Title/Abstract]) OR (Smoking Habit[Title/Abstract]) OR (Habit, Smoking[Title/Abstract]) OR (Habits, Smoking[Title/Abstract]) OR (Smoking Habits[Title/Abstract]))) AND (rupture[Title/Abstract]). The title and abstract of each study were independently screened by two researchers trained in standardization and uniformity. After our initial screening, we obtained the full text of all studies that were likely to meet our minimum inclusion criteria.

### Data Extraction and Quality

A systematic review and a meta-analysis were performed according to the Preferred Reporting Items for Systematic Reviews and Meta-Analyses (PRISMA) guidelines. Two evaluators independently assessed the quality of all included studies using the nine-star Newcastle-Ottawa Scale (NOS). The studies were scored according to the three aspects of the NOS assessment: selection, comparability, and outcome. Studies with NOS scores≥6 are considered to be of high quality. A reviewer extracts all study data. All disagreements were discussed, and a final decision by the two reviewers was obtained. The characteristics and scores of the studies included in this meta-analysis are shown in Tables 1, 2. We identified former smokers as quitters.

**Table 1:** The characteristics of the studies included in this meta-analysis.

**Study (Author, years)**	**Study design**	**participants**	**Age (mean ±SD)**	**Female (*n*%)**	**Smoking status**	**Smoking rate**	**Rupture rate**	**Missing data**	**NOS score**
Xu (13)	Retrospective study	112	39 ± 6	69.6%	Current smoking	26.7%	61.6%	0	6
Wang (14)	Retrospective study	235	—	51.4%	Current smoking	38.7%	75.3%	0	6
Peng (15)	Retrospective study	291	—	70.7%	Current smoking	51.5%	70.4%	0	6
Li (16)	Retrospective study	474	57	58.6 %	Current smoking	20.4%	64.8 %	0	6
Zheng (17)	Retrospective study	200	47.9 ± 3.3	62%	Current smoking	19%	79%	0	6
Liu (18)	Retrospective study	594	56.1 ± 12.3	47.1%	Current smoking	30.6%	90.9%	0	6
Ho et al. (19)	Retrospective study	199	53.8 ± 12.4	77.3%	Current smoking	81.5%	58.7%	0	7
Vlak (20)	Retrospective study	456	—	71.5%	Current smoking	55%	54.8%	0	6
Jiang (21)	Retrospective study	97	—	74.2%	History of smoking	24.7%	59.7%	0	6
Feng et al. (22)	Retrospective study	385	55.9 ± 9.3	100%	History of smoking	7%	22.5%	0	7
Feng et al. (10)	Retrospective study	381	—	58.2%	History of smoking	30.9%	33.3%	0	7
Juvela et al. (23)	Prospective study	142	—	53.5%	History of smoking	60.5%	23.9%	0	6
Ma (24)	Retrospective study	103	55.4 ± 12.31	61.1%	Current smoking	34.9%	57.2%	0	6
Can et al. (25)	Retrospective study	4701	55.6	77.9%	History of smoking	55.4%	27.7%	210	6

**Table 2:** NOS scores for included studies.

	**Selection**				**Comparability**		**Exposure**			
**Study (author/year)**	**A**	**B**	**C**	**D**	**E**	**F**	**G**	**H**	**I**	**NOS Score**
Xu (13)	1	1		1			1	1	1	6
Wang (14)	1	1		1			1	1	1	6
Peng (15)	1	1		1			1	1	1	6
Li (16)	1	1		1			1	1	1	6
Zheng (17)	1	1		1			1	1	1	6
Liu (18)	1	1		1			1	1	1	6
Ho et al. (19)	1	1		1	1		1	1	1	7
Vlak (20)	1	1		1			1	1	1	6
Jiang (21)	1	1		1			1	1	1	6
Feng et al. (22)	1	1		1	1		1	1	1	7
Feng et al. (10)	1	1		1	1		1	1	1	7
Juvela et al. (23)	1	1	1			1	1	1		6
Ma (24)	1	1		1			1	1	1	6
Can et al. (25)	1	1		1	1		1	1		6

*A: case definition and diagnosis/exposure cohort representative. B: case representativeness/selection of non-exposed cohorts. C: control selection/exposure determination. D: definition of control/ demonstration that outcome of interest was not present at start of study. E and F: case and control comparability/comparability of exposed and non-exposed cohorts. G: survey and evaluation methods/outcome determination. H: cases and controls were surveyed in the same way/adequate follow-up time. I: same non-response rate/completeness of follow-up*.

### Statistical Analysis

In this meta-analysis, the association between smoking status and IA was measured by estimating the relative risk (RR) with its 95% confidence interval (CI). Statistical heterogeneity was assessed using *I*^2^, and if *I*^2^ is more than 50% was analyzed using a random-effects model, and a sensitivity analysis was further performed. if *I*^2^ is <50% was analyzed using a fixed-effects model, and no sensitivity analysis was performed. Differences were considered significant if two-sided *p* < 0.05.

## Results

### Study Selection and Characteristics

**Figure 1:**
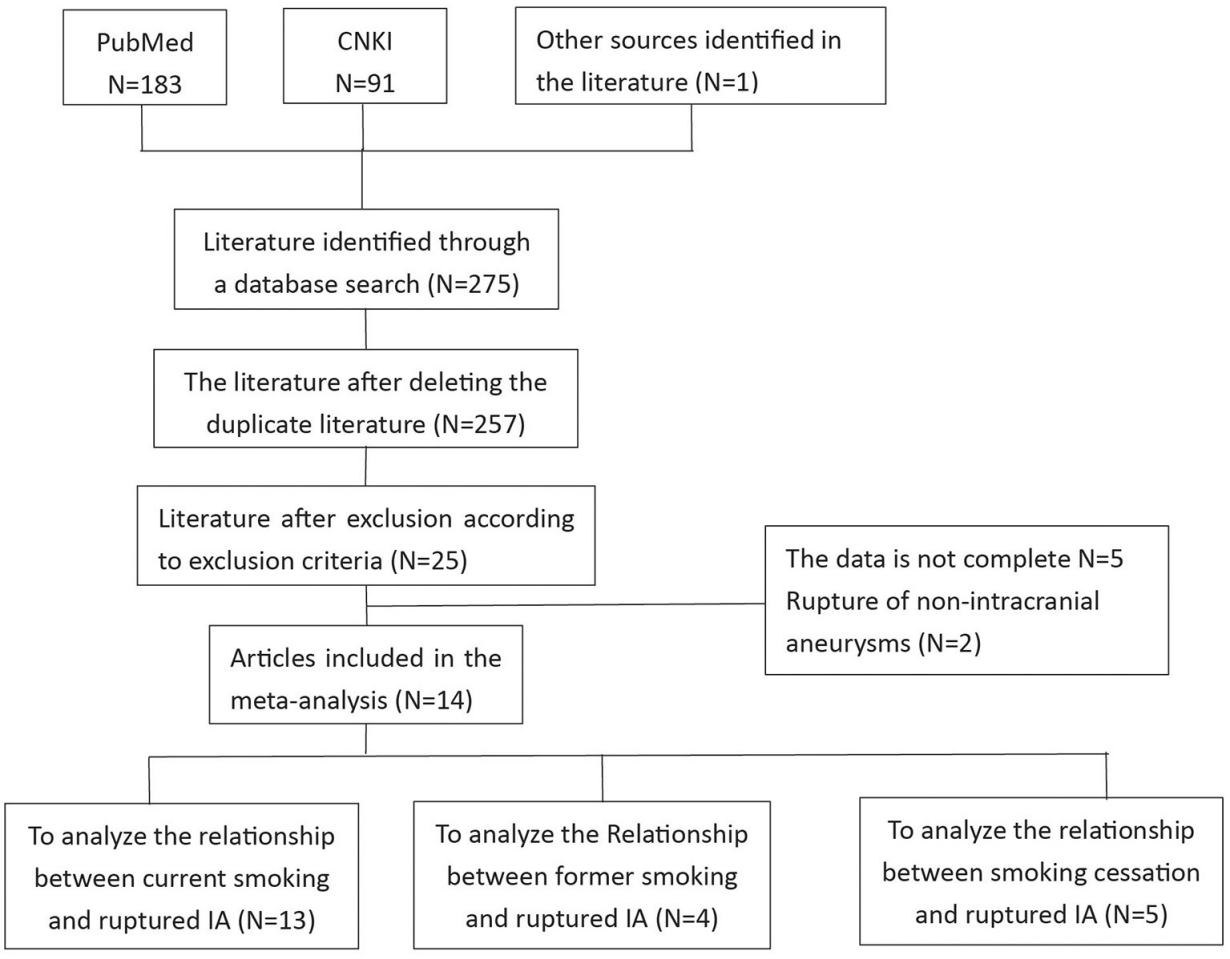
A flow chart of literature selection.

We found a total of 275 articles. After the elimination of duplicate literature, the literature that did not meet the inclusion criteria were excluded through preliminary screening of the title and abstract. There were 25 pieces of literature remaining, and the applicability of these 25 pieces of literature was evaluated by reading the full text. After the articles were further excluded according to the exclusion criteria, there were still 14 articles that met the inclusion criteria. These 14 articles were included in the meta-analysis. The flow chart of literature screening is shown in Figure 1. These 14 articles included 13 retrospective studies and 1 prospective study. A total of 6,196 patients were included in the 14 articles, including 2,558 current smokers, 1,216 former smokers, 2,422 non-smokers, and 3,014 ruptured IA. The characteristics of the studies included in this meta-analysis are shown in Table 1.

### Association Between Smoking Status (Current Smoking, Former Smoking, and Non-Smoking) and Ruptured IA Estimated With Relative Risk

**Figure 2:**
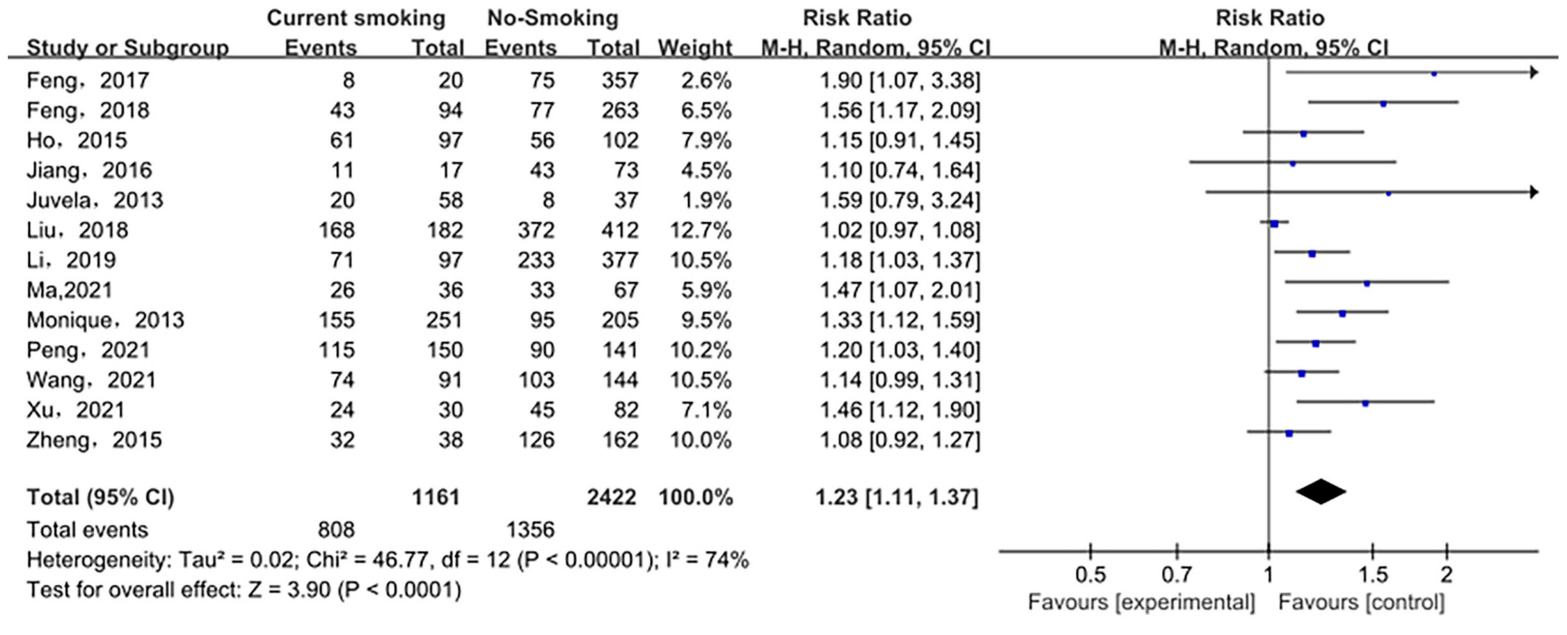
A Forest plot of the current smoking and non-smoking groups.

**Figure 3:**
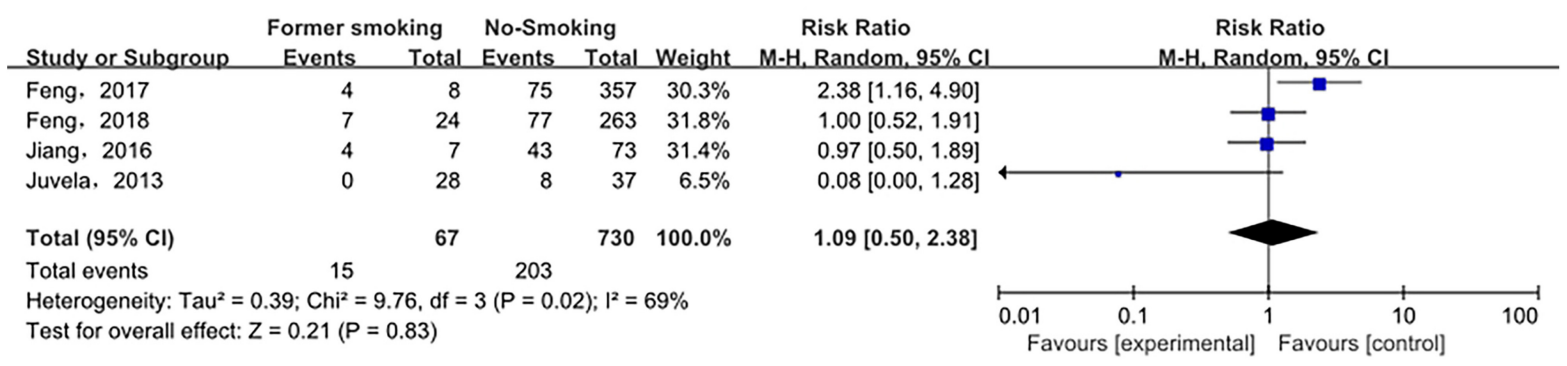
A Forest plot of the former smoking and non-smoking groups.

**Figure 4:**
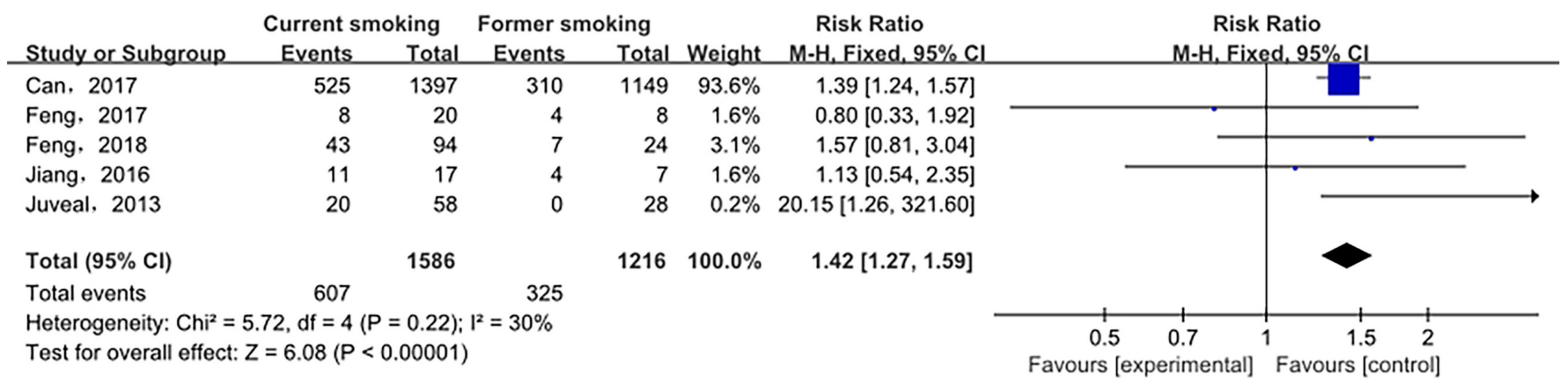
A Forest plot of the current smoking and the former smoking groups.

The relationship between smoking status (current smoking, former smoking, and non-smoking) and ruptured IA was evaluated based on relative risk. The risk of ruptured IA in the current smoking group was significantly higher than that in the non-smoking group, with statistical significance (RR_total_ = 1.23, 95%*CI*: 1.11–1.37). The analysis was estimated using a random-effects model because significant heterogeneity was found between studies (*p* = 0.00001 < 0.05, *I*^2^ = 74% > 50%). These data are shown in Figure 2. There was no significant difference in the risk of ruptured IA between the former smoking (smoking cessation) group and the non-smoking group (RRtotal = 1.09, 95%*CI*: 0.50–2.38). The analysis was estimated using a random-effects model because significant heterogeneity was found between studies (*p* = 0.02 < 0.05, *I*^2^ = 69% > 50%). These data are shown in Figure 3. The risk of the ruptured IA in the current smoking group was significantly higher than that in the former smoking (smoking cessation) group, with a statistically significant difference (RRtotal = 1.42, 95%*CI*: 1.27–1.59). Since there was no significant heterogeneity between the studies, the fixed-effects models were used to estimate the analyses (*p* = 0.22 > 0.05, *I*^2^ = 30% < 50%). These data are shown in Figure 4.

### Sensitivity Analysis and Publication Bias

**Figure 5:**
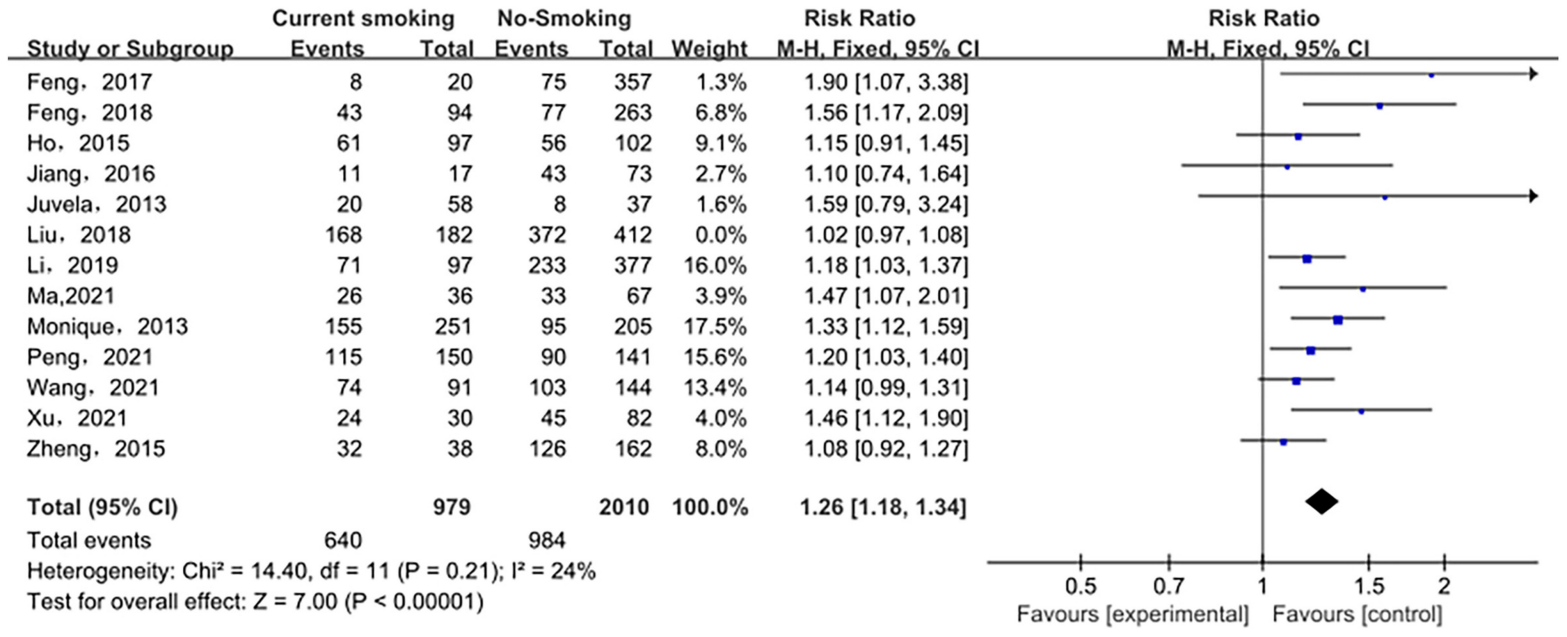
A Forest plot of the current smoking and non-smoking groups after heterogeneous cohorts were removed by sensitivity analysis.

**Figure 6:**
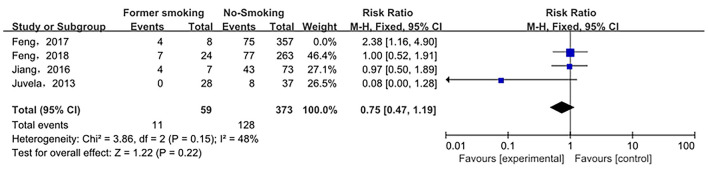
A Forest plot of the former smoking and non-smoking groups after heterogeneous cohorts were removed by sensitivity analysis.

**Figure 7:**
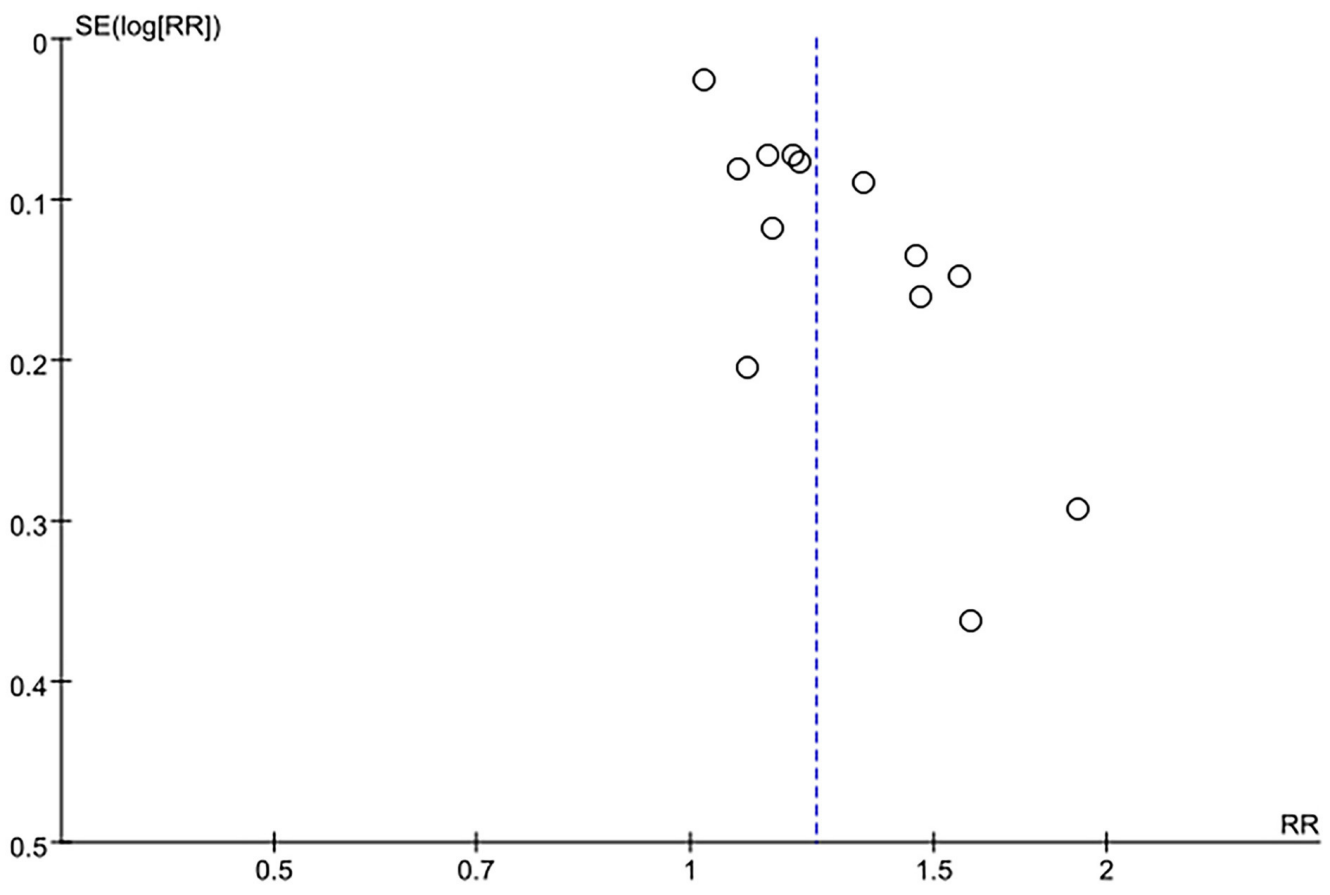
A Funnel plot of the current smoking and non-smoking groups.

**Figure 8:**
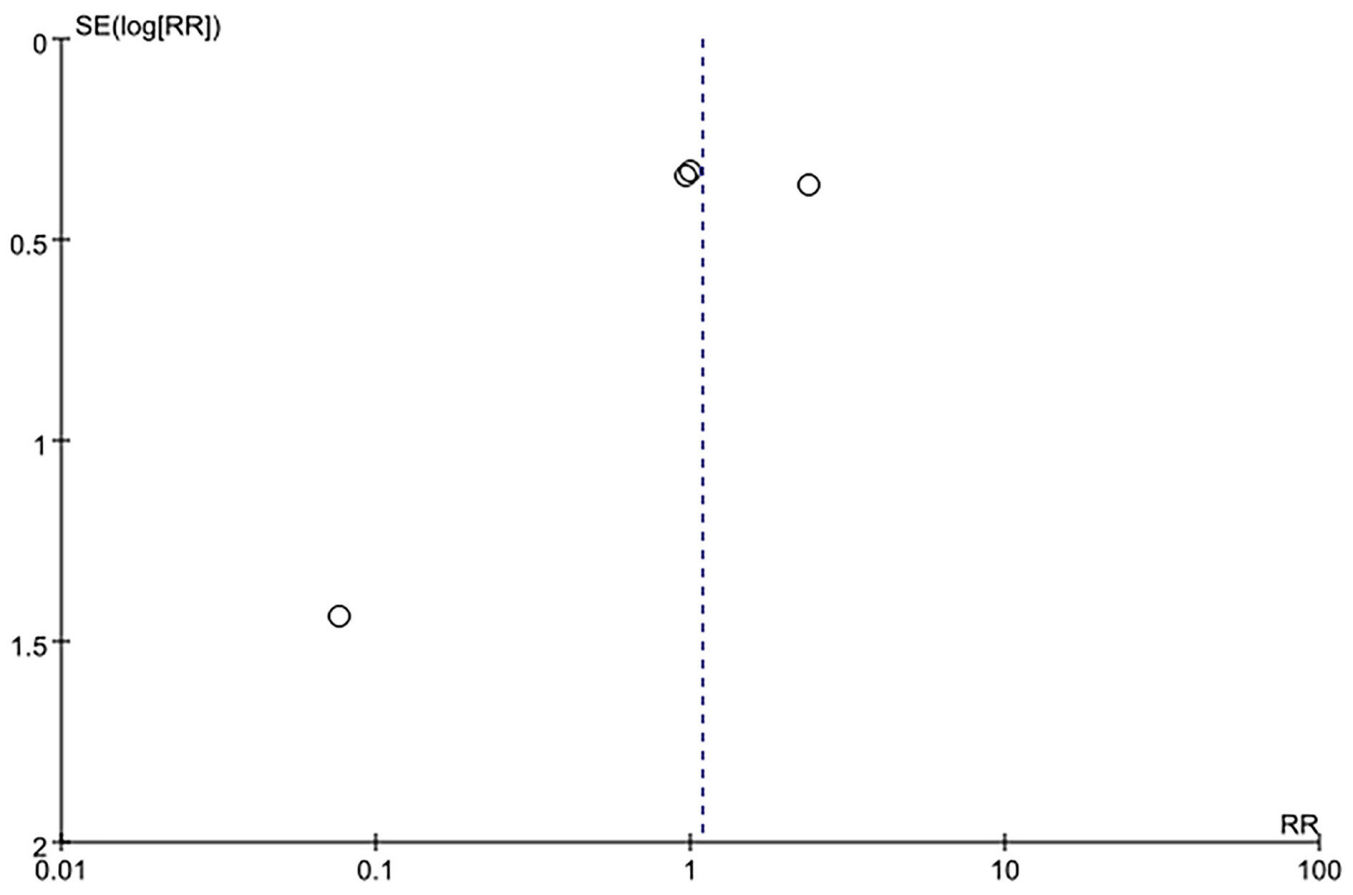
A Funnel plot of the former smoking and non-smoking groups.

**Figure 9:**
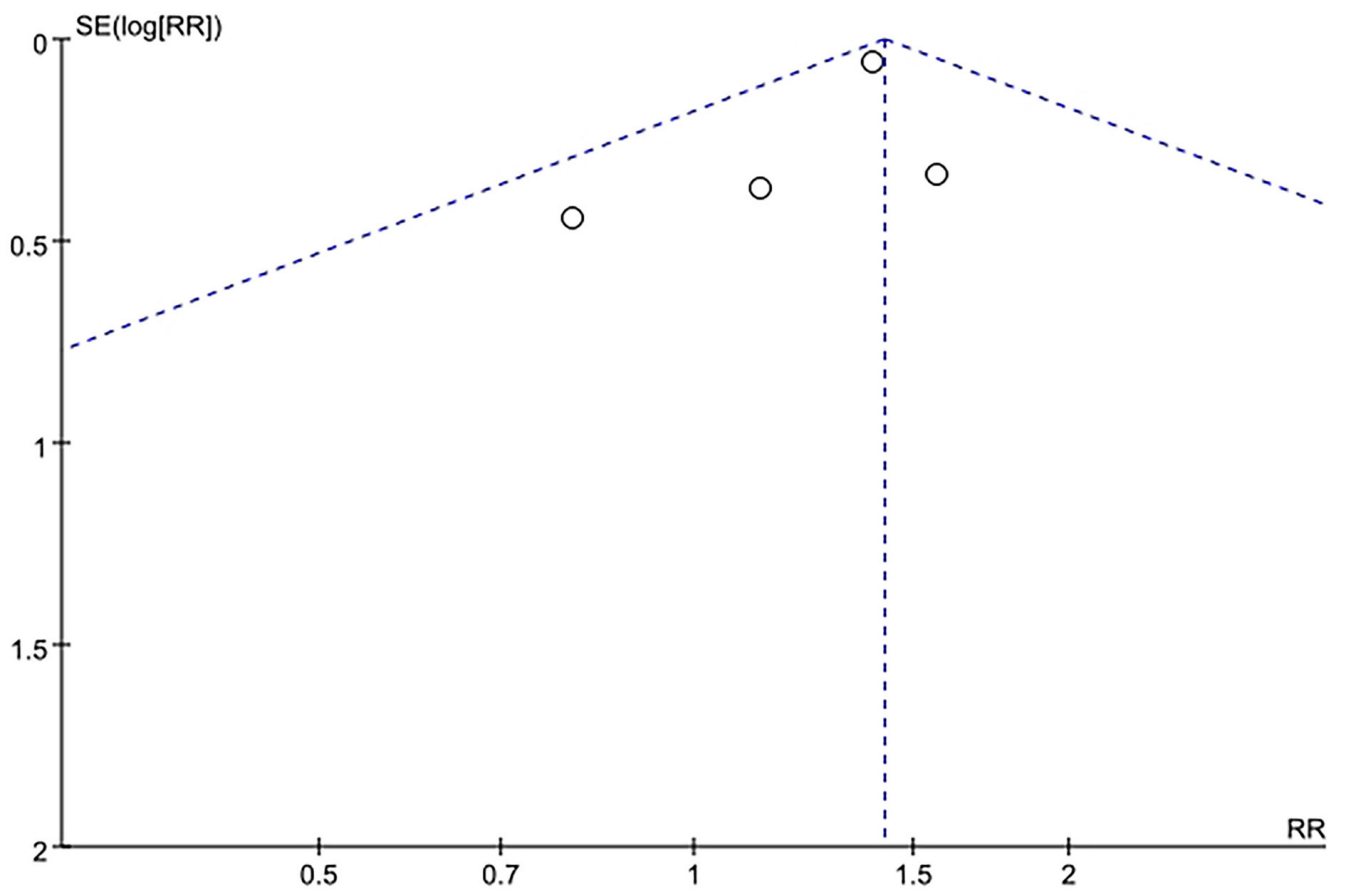
A Funnel plot of the current smoking and the former smoking groups.

Sensitivity analysis of the current smoking group and the non-smoking group found that Liu's study data were the main source of heterogeneity. These studies were removed, and there was no statistical heterogeneity among these cohorts (*p* = 0.21> 0.05, *I*^2^ = 24% < 50%). This meta-analysis, again calculated using the fixed-effect model, shows that current smoking remains a risk factor for ruptured IA (RRtotal = 1.26,95%*CI*: 1.18–1.34) (Figure 5). Sensitivity analysis of the former smoking group and the non-smoking group found that Feng's et al. study (2017) data were the main source of heterogeneity. These studies were removed, and there was no statistical heterogeneity among these cohorts (*p* = 0.15 > 0.05, *I*^2^ = 48% <50%). This meta-analysis, again calculated using the fixed-effects model, showed no statistical difference in the risk of ruptured IA between the former smokers and the non-smokers (RRtotal = 0.75, 95%*CI*: 0.47–1.19) (Figure 6). Potential publication bias was visually assessed using funnel plots generated in RevMan. However, the small amount of study data limits the interpretability of the findings, as shown in Figures 7–9.

## Discussion

Previous studies have documented that smoking is one of the most important risk factors for the formation of IA. However, there are still several studies with controversial results. We have presented an integrated overview and a systematic review of recent studies on the relationship between the current smoking, the former smoking (smoking cessation), and the non-smoking groups and the risk of ruptured IA. Further, we discussed the underlying mechanism of smoking on IA formation and rupture.

The global decline in the incidence of SAH parallels the decline in smoking prevalence. For every 1% point decrease in the smoking rate, the overall age- and gender-adjusted SAH incidence decreases by 2.4% (26), which clearly illustrates that the smoking is related to ruptured aneurysms. Moreover, genetic susceptibility to smoking leads to an increased risk of IA and drives most of the genetic association between IA and other cerebrovascular traits (27, 28). To better understand the relationship between smoking and ruptured IA, scholars analyzed the relationship between smoking time, smoking intensity, smoking index, and smoking status and ruptured IA. One case-control study showed that current smoking, smoking intensity, and smoking duration are significantly related to ruptured IA (25). There is also a dose-response relationship between the intensity, duration of smoking, and the incidence of ruptured IA (10). As the number of cigarettes smoked per day (CPD) and the number of years of smoking increased, the risk of IA also increased accordingly (25). Moreover, a positive correlation between the smoking index (CPD × years of tobacco use) and the risk of ruptured aneurysms were also reported (10). According to published data, current smokers are 2.5 times more likely to have a rupture than non-smokers, while former smokers are nearly 2 times more likely to have a rupture than non-smokers (25, 29). Compared with former smokers and non-smokers, current smokers are more likely to develop ruptured aneurysms, which is consistent with our results. Prospective studies showed that the risk of ruptured aneurysms will be reduced when patients quit smoking (12, 30). However, the study of Anil and his colleagues reported that the longer the smoking cessation period, the lower the risk of ruptured aneurysms in former smokers, but this association disappeared after adjusting for other confounding factors in the multivariate analysis (25). This indicates that the risk of ruptured IA caused by smoking could still exist even after smoking cessation. The reason for this phenomenon might be that smoking has caused irreversible damage. For example, the nicotine in cigarettes damages elastin, which has a low repair potential (31). Therefore, the elastin damage caused by long-term smoking may be permanent, even after smoking cessation. In addition, for a long time after quitting smoking, quitters showed persistent low-grade inflammation (32). These results obtained from Can et al. showed that smoking cessation only reduces the cumulative dose of tobacco in the body and that early quitting does not reduce the risk of SAH. This view has sparked debate about the relationship between smoking cessation and the risk of ruptured IA. Then, we further evaluated the association between smoking cessation (Former smoking) and the risk of ruptured IA. The results showed that there was no significant difference in the risk of ruptured IA between the former smoking (smoking cessation) group and the non-smoking group, suggesting that quitting smoking may be beneficial for patients with unruptured IA. Moreover, smoking cessation can also reduce the damage of delayed cerebral ischemia after SAH. Delayed cerebral ischemia is the leading cause of death and disability after ruptured IA (33). Therefore, smoking cessation is still indispensable in the treatment of IA. Moreover, those results emphasized the importance of quitting smoking in patients with unruptured IA.

The majority of previous research has focused on the effects of active smoking on IA. In contrast, people's understanding of the effects of passive smoking on IA is still incomplete. The two primary sources of exposure to passive smoking are the workplace and the home (such as a smoking spouse), and passive smoking is common among women. Previous studies have shown that, in non-smoking women with IA, passive smoking is not an independent risk factor for ruptured IA (10). However, passive smoking reduces eNOS activity and increases vascular endothelial inflammation similar to active smoking, which indicates that passive smoking has direct damage to the vascular system (34). There seems to be no harm in avoiding passive smoking in one's everyday life, although the association between passive smoking and the formation and rupture of IA remains indistinct. Further, we summarized the differences in aneurysm morphology between smokers and non-smokers (Table 3), and some aneurysm morphology parameters were significantly correlated with smoking status, such as larger daughter vessel diameters, larger size ratio, and the location at the basilar apex (19). Compared with non-smokers, current smokers may have larger daughter vessel diameters and size ratio, and smokers are more likely to develop basilar apex aneurysms (19). The size ratio is the ratio of the maximum aneurysm height to the mean diameter of the artery bearing the aneurysm (37). The larger size ratio means that large aneurysms formed by small blood vessels are more likely to rupture than small aneurysms formed by large blood vessels. Aneurysms at the Basilar apex have a higher risk of rupture, morbidity, and mortality (38, 39). Of course, the mechanism of smoking-induced changes in morphological parameters of these IA is indistinct, which requires further study. The morphological changes of the IA can reflect the process of its occurrence, growth, and rupture. The relationship between smoking and these specific morphological changes could provide a morphological basis for how smoking increases in aneurysm formation and rupture. However, not all morphological parameters of IA are related to smoking. The study by Juchler et al. found no association between the luminal shape of the IA and the patient's sex, age, smoking, or history of hypertension, even though these factors were strongly associated with aneurysm incidence (28, 40).

**Table 3:** Relationship between smoking and intracranial aneurysm rupture and intracranial aneurysm morphology.

**Smoking status / Morphological parameters of intracranial aneurysms**	**Risk of ruptured intracranial aneurysm**	**Main reported findings**	**References**
Current smoker	Increased risk of rupture	Current smokers had a noticeable growth risk of ruptured aneurysms compared with never-smokers or former smokers.	(10)
Former smoker	Increased risk of rupture	Former smokers had a significantly increased risk of aneurysm rupture compared with those who had never smoked.	(25)
Non-smokers	risk-free	Smoking is an independent risk factor for intracranial aneurysm formation and rupture.	(35)
Passive smoking	risk-free	Passive smoking is not an independent risk factor for intracranial aneurysm rupture.	(36)
Smoking cessation	Reduce the risk of rupture	Patients with intracranial aneurysms have a reduced risk of rupture after quitting smoking.	(12, 30)
larger size ratio, Aneurysms at the Basilar apex	Increased risk of rupture	Compared with non-smokers, current smokers had a larger size ratio of intracranial aneurysms and were more likely to have basilar apex aneurysms.	(19)

*Current smoker: patients who smoked at the time of treatment or smoked ≥100 cigarettes during the past year (10). Former smoker: patients who quit smoking at least 1 year before diagnosis of IA (25)*.

**Figure 10:**
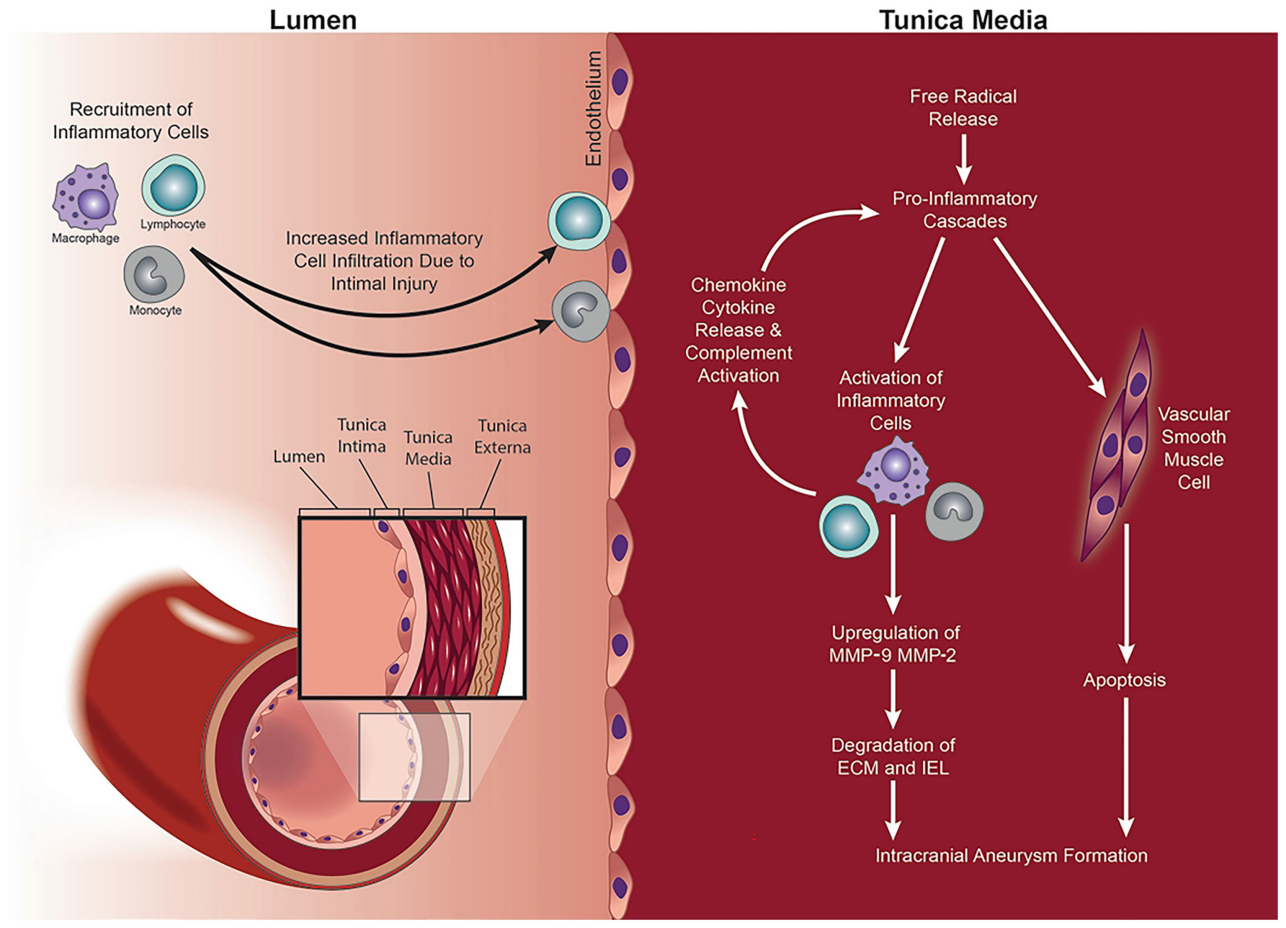
MMPs mediate IA formation via a process of vascular remodeling. Intimal injury and increased wall shear stress (WSS) lead to the recruitment and infiltration of inflammatory cells into the vessel wall. Release of free radicals, pro-inflammatory chemokines and cytokines, and activation of the complement cascade mediate the upregulation of MMP-2 and MMP-9. The consequent proteolytic process results in the degradation of the ECM within the tunica media and altered the structure of the vessel wall with IA formation. Copyright Department of Neurosurgery, The University of Utah. Published with permission (44).

**Figure 11:**
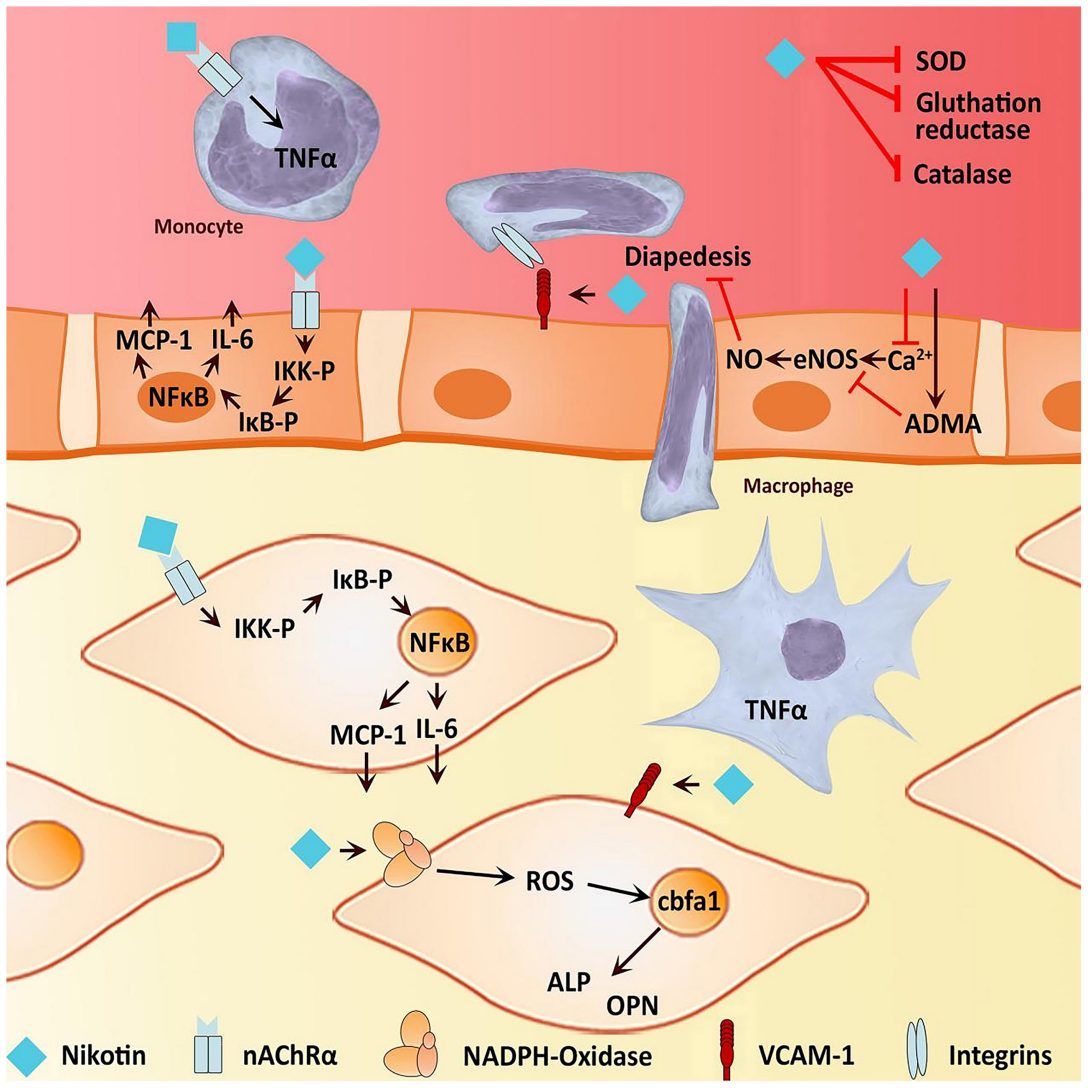
Effects of nicotine on osteogenic transdifferentiation. Nicotine could induce an environment that promotes or even initiates vascular calcification of the media vessel wall by osteogenic transdifferentiation of VSMC. On the one hand, nicotine is capable of inducing inflammation by stimulating the activity of NF-κB and therefore the expression of pro-inflammatory cytokines such as IL-6. Furthermore, there is evidence for nicotine to stimulate macrophages to express TNF-α, contributing to an inflammatory environment. On the other hand, nicotine increases oxidative stress, directly and indirectly, possibly leading to endothelial dysfunction Both endothelial dysfunction and an inflammatory environment facilitate an osteogenic phenotype of VSMC (45).

It is well-known that IA formation is associated with hemodynamics, chronic inflammation, vascular endothelial dysfunction, and vascular remodeling (41–43) (Figure 10). Pieces of evidence have shown that smoking could induce an inflammatory response in blood vessels, hemodynamic stress, endothelial dysfunction, and eventually cause weakened blood vessel walls and rupture of blood vessel walls (25, 34, 44) (Figure 11). Smoke mainly contains chemicals such as tar, carbon monoxide, nicotine, nitrosamines, volatile organic compounds, and polycyclic aromatic hydrocarbons, which are characteristic of tobacco (46). Nicotine is a major biologically active constituent of tobacco products, which has deleterious effects on IA formation, and rupture (Table 4). Nicotine is also one of the most significant components that cause vascular inflammation (59). The early and key step of cigarette smoke-induced vascular inflammation is the activation of the NF-κB pathway (52), which might be related to nicotine (46). Nicotine also directly stimulates macrophages to secrete high levels of inflammatory factors such as TNF-α, IL-1β, and other chemokines (53). Moreover, the nicotine-induced endothelial dysfunction and inflammatory microenvironment also promote vascular smooth muscle cells (VSMCs) osteogenic transdifferentiation and vascular wall calcification. Experiments have shown that chronic nicotine exposure induces the expression of MMPs in the arterial wall and the destruction of the arterial wall structure (elastin damage) (54). Long-term smoking can impair vascular endothelial function (60), which may be also related to nicotine and oxidants in cigarette smoke. Nicotine promotes oxidative stress in VSMCs and endothelial cells by downregulating superoxide dismutase (SOD), catalase, and glutathione reductase (45). Together, smoking exposure could cause endothelial dysfunction, increase oxidative stress, and increase morbidity and mortality of cardiovascular diseases, which promotes the formation and rupture of IA. Therefore, to prevent the growth and rupture of IA, we underline that patients necessarily cease smoking, which is a valuable measure.

**Table 4:** Nicotine may affect the pathological mechanism of intracranial aneurysms.

**Possible effects of nicotine**	**Influenced process**	**Pathological mechanism of intracranial aneurysm**	**References**
Binding to the α3β4 nAChRs^a^ results in the release of catecholamines.	Increased heart rate, increased blood pressure, increased cardiac output	Hemodynamics	(47–49)
Promote fat breakdown and increase FFA^b^ levels	Dyslipidemia, Blood viscosity increases	Hemodynamics	(50)
Reduced activity of eNOS^c^	Vascular endothelial dysfunction	Vascular endothelial dysfunction	(51)
Inhibit SOD^d^ catalase and glutathione reductase	Oxidative stress of VSMCs^e^ and endothelial cells	Vascular endothelial dysfunction and inflammation	(45)
Activation of NFκB-pathway and inflammatory mediators	Inflammation	Inflammation	(45, 52, 53)
Increased expression of MMP-2 and MMP-9^f^	Extracellular matrix degradation	Vascular remodeling	(54–57)
Induce the influx of calcium ions in VSMCs	VSMCs change from contractile to synthetic	Vascular remodeling	(58)

a *nAChRs, nicotinic acetylcholine receptors*.

b *FFA, free fatty acid*.

c *eNOS, endothelial nitric oxide synthase*.

d *SOD, superoxide dismutase*.

e *VSMCs, vascular smooth muscle cells*.

f *MMP, matrix metalloproteinase*.

There are some limitations to this study. First, all the included studies are observational, and inevitably, there is an observational bias. Second, different studies use different criteria to define current smoking and former smoking. In addition, we did not consider gender, age, race, hypertension, and other factors in the study. Subgroup analyses were not performed because we could not extract relevant data for some factors from the included literature, which may increase heterogeneity among the included studies. These unspecified factors and other unmeasured or unknown confounding factors can also affect the reliability of these findings. We divided tobacco exposure into current smoking, former smoking, and non-smoking. To some extent, this may reduce heterogeneity between included studies. Finally, due to the relatively small number of studies included, the statistical power of these results is limited.

A larger and prospective cohort study should be conducted to validate our results in the future.

In conclusion, there is a dose-response relationship between the intensity and duration of smoking and the increased risk of ruptured IA. In terms of the morphology of IA, smoking causes IA to form morphologies that are more prone to rupture. The risk of ruptured IA was not statistically different between the former smoking (smoking cessation) group and the non-smoking group. To summarize, smoking is involved in the formation and rupture of IA, and it is indispensable for patients with IA to quit smoking. Despite numerous related studies on the formation and rupture of IA, the mechanism of IA formation and rupture is still inaccurate, which requires further investigation in our future.

## Author Contributions

CL and LZ: conception, design, and critically revising the article. HW, LW, and JW: acquisition of data. HW and LW: drafting the article. All authors reviewed submitted version of manuscript.

## Funding

This work was supported by the Scientific Research Starting Foundation of Affiliated Hospital of Hebei University [Grant Number 31010413]; the Provincial Medical Talents Project funded of Hebei Province [Grant Number 361007]; the Key Scientific Research Projects of the Affiliated Hospital of Hebei University [Grant Number 2021Z001].

## Conflict of Interest

The authors declare that the research was conducted in the absence of any commercial or financial relationships that could be construed as a potential conflict of interest.

## Publisher's Note

All claims expressed in this article are solely those of the authors and do not necessarily represent those of their affiliated organizations, or those of the publisher, the editors and the reviewers. Any product that may be evaluated in this article, or claim that may be made by its manufacturer, is not guaranteed or endorsed by the publisher.

## Abbreviations

IA: intracranial aneurysm
SAH: subarachnoid hemorrhage
WSS: wall shear stress
MMPs: matrix metalloproteinases
VSMCs: vascular smooth muscle cells
MCP1: macrophage chemotactic protein 1.
